# A new wireless system for decentralised measurement of physiological parameters from shake flasks

**DOI:** 10.1186/1475-2859-5-8

**Published:** 2006-02-24

**Authors:** Antti Vasala, Johanna Panula, Monika Bollók, Lutz Illmann, Christian Hälsig, Peter Neubauer

**Affiliations:** 1 Bioprocess Engineering Laboratory, Department of Process and Environmental Engineering and Biocenter Oulu, University of Oulu, Oulu, Finland; 2 teleBITcom GmbH, Teltow, Germany

## Abstract

**Background:**

Shake flasks are widely used because of their low price and simple handling. Many researcher are, however, not aware of the physiological consequences of oxygen limitation and substrate overflow metabolism that occur in shake flasks. Availability of a wireless measuring system brings the possibilities for quality control and design of cultivation conditions.

**Results:**

Here we present a new wireless solution for the measurement of pH and oxygen from shake flasks with standard sensors, which allows data transmission over a distance of more than 100 metres in laboratory environments. This new system was applied to monitoring of cultivation conditions in shake flasks. The at-time monitoring of the growth conditions became possible by simple means. Here we demonstrate that with typical protocols *E. coli *shake flask cultures run into severe oxygen limitation and the medium is strongly acidified. Additionally the strength of the new system is demonstrated by continuous monitoring of the oxygen level in methanol-fed *Pichia pastoris *shake flask cultures, which allows the optimisation of substrate feeding for preventing starvation or methanol overfeed. 40 % higher cell density was obtained by preventing starvation phases which occur in standard shake flask protocols by adding methanol when the respiration activity decreased in the cultures.

**Conclusion:**

The here introduced wireless system can read parallel sensor data over long distances from shake flasks that are under vigorous shaking in cultivation rooms or closed incubators. The presented technology allows centralised monitoring of decentralised targets. It is useful for the monitoring of pH and dissolved oxygen in shake flask cultures. It is not limited to standard sensors, but can be easily adopted to new types of sensors and measurement places (e.g., new sensor points in large-scale bioreactors).

## Background

Shake flask cultivation is an easy way to grow small amounts of microbes. They are also applied for bioprocess optimization and for preparation of starter cultures for bioreactor cultivations. Shake flask cultures are usually batch-cultivations since the media components are added into the cultivation flask already at the beginning. In aerobic cultures, due to non-limited growth anaerobic conditions emerge easily because shaking can not provide enough oxygen for fast-growing microbes in dense cultures. In contrast growth can be controlled by substrate limitation in bioreactor cultivations. The availability of high concentration of glucose in shake flask enhances synthesis of overflow metabolites, since the capacities of the respiratory system and citric acid cycle can not process all the glucose taken into the cell. Generally, cell and product yields may vary very much between shake flask cultivations. The reasons for poor reproducibility can not be recognised without monitoring.

However, especially for the cultivation of starter cultures the quality of the culture is of crucial importance; the accumulation of harmful metabolites during pre-cultivation, starvation, anaerobiosis and change of the pH level retard the growth of microorganisms in the main bioreactor cultivation. Non-optimal conditions may favour faster growing mutants, such as recombinant constructs that have aborted the plasmid, and evoke expression of stress-related genes [1]. Well-equipped systems of small parallel bioreactors have been developed [2], but they generally lack the flexibility of shake flask cultures. Improvement in shake flask culture quality can be obtained with small changes. For example shake flask design and plug material affect the oxygen transfer rate [3,4]. Oxygen consumption can be reduced by the use of lower cultivation temperature or by limiting substrate feeding [5,6]. Acidification of the medium may be minimised by selecting a suitable host strain [7] or cultivation medium. Oxygen sensors would provide information of oxygen-limitation or starvation (recognised as a sudden increase of oxygen level due to decreased respiratory activity), but they are usually not available for shake flask conditions. Since the possibilities for pH control, substrate feeding or oxygen supply are limited in shake flasks, the design of cultivation conditions is usually done before according to the researcher's experience. Most often this experience is obtained by trial and error and by performing parallel cultivations in different conditions. Measuring possibilities would greatly speed up this development process.

Only few commercial solutions for small size bioreactor systems have been presented so far [8]. The Dasgip (Dasgip AG, Jühlich, Germany) system contains a battery of small bioreactor vessels. The PreSens system (Precision Sensing GmbH, Regensburg, Germany) provides systems for both oxygen level and pH measurement from the culture. Ramos (respiration activity monitoring system, AC Biotech, Germany) is designed for measurement of oxygen and carbon dioxide from shake flasks [2]. The Bluesens system (BlueSens GbR, Germany) offers oxygen and CO_2 _gas sensors (and their adapter) for shake flasks, which however due to their size and weight are only applicable to larger shake flasks. Despite the possibilities to use small size bioreactors, even microtiter plates, the above mentioned systems are not fully flexible. They are not cheap solutions, because special sensors or reader units must be purchased.

### Sensor systems and measurement platforms applicable to small scale cultivations

For high-throughput screening purposes the tendency towards miniature size bioreactors continues [9-14]. Miniature size bioreactors can not fully replace the use of shake flasks, since they usually are even more limited in view of measurement and control. Additionally their characterisation is more complicated by the limited amount of samples which can be withdrawn and volume changes due to evaporation so that still about 90 % of all small scale cultivations are performed in shake flasks [8]. However, emerging developments in miniaturised sensors, such as fiber-optic chemical pH sensors, fluorescence detector systems which can be used for oxygen level monitoring in shake flasks [15-17] are highly valuable tools for microtiter plate cultivations [10,18-20]. Such fluorescent sensor systems may tolerate several autoclavation cycles [21], are scaleable and available for several cultivation platforms, but they require fibre optics and fluorescent readers.

Despite and because of these fascinating developments of new biosensors, we have realised a need for a technical platform which supports several sensor types and is applicable in shake flasks, without the need for reconstruction of existing shaking devices. Here we present a new technical system which provides new flexibility of introducing sensors into shake flask and fermenter cultures by the use of wireless technology which removes the need for cable connections. This system is a modular platform, which supports several sensor types and computer instead of separate measuring devices for centralized data collection and management.

In this paper we demonstrate for the advances of the SENBIT technology for monitoring physiological events in shake flasks at the example of pH and oxygen changes in *E. coli *cultures and the possibilities for process optimisation at the examples of the critical feeding of methanol in cultures of *Pichia pastoris*.

## Results and discussion

### SENBIT – a new wireless system for use of sensors in shake flasks

**Figure 1:**
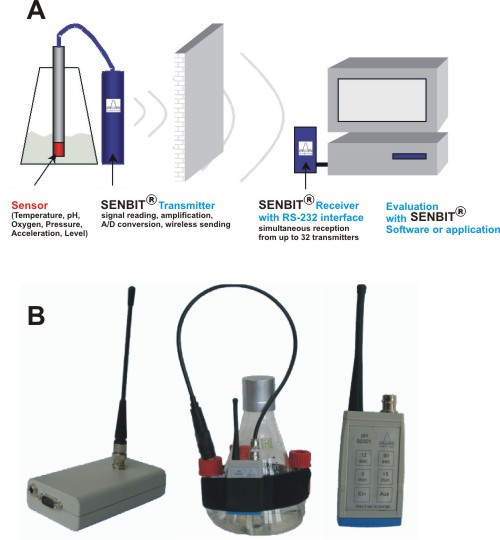
**SENBIT system**. A) operation principle B) SENBIT device (receiver, shake flask system and transmitter).

As the use of standard sensors and measuring devices is not practical for shake flask cultivations which are performed in diverse places of an institution, we aimed in developing a wireless modular platform. This platform should be applicable to parallel monitoring of different cultures and should not be limited to a certain kind of sensor. The solution is a wireless, modular platform which uses the ISM band of 433 MHz to send digitalized data from sensors directly to a computer. This band is able to penetrate solid barriers (like concrete or brick walls). Therefore a decentralized location of measurement points even inside buildings and incubators is possible. In open space, reading from over 400 m distance can be achieved. For normal laboratory conditions, regular readings are realised from up to 100 m distance, including closed incubation chambers or incubation rooms. This solution substitutes separate measurement devices and long cables between sensors and devices. Each sensor is directly attached to a transmitter (Fig. 1), which reads and amplifies the electrical signal coming from a sensor, converts the signal to a digital form and sends the measurement value and transmitter ID-code within the 433 MHz band-width to the receiver unit. The receiver is connected to a computer via an RS-232 or to an USB port via an RS-232/USB adapter. The developed software runs under Microsoft Windows (98, NT4, 2000, XP). The researcher can define sensor types, calibration procedures can be performed and new projects can be created. Furthermore it is possible to measure values, and perform data reporting as well as data manipulation (removal of outline values, reduction of values). The new platform supports currently up to 32 sensors but can be extended if needed. Several types of sensors can be used at the same time, because the digitalized data sent by sensor/transmitter are interpreted and converted to measuring values by the computer. This implies that several research experiments can run at the same time.

### What happens in a typical *Escherichia coli *shake flask cultivation?

**Figure 2:**
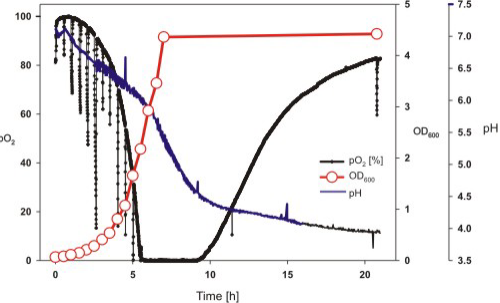
**pH and dissolved oxygen tension in a typical *E. coli *shake flask cultivation measured with SENBIT^®^**. The short periods of oxygen decrease are caused by stops of the shaker due to sampling. Strain RV308 was cultivated in mineral salt medium (baffled 1 l shake flask with 200 ml medium, 37°C, 180 rpm shaking, antifoam 0.1 ml l^-1^).

The SENBIT system was used to measure pH and oxygen saturation levels of *Escherichia coli *cultures in shake flasks. Biomass growth was monitored by measuring the optical density at 600 nm. As shown in Fig. 2, non-controlled shake flask cultures will inevitably suffer from oxygen limitation and acidification of the medium. Oxygen limitation (after 5 h) seems to enhance accumulation of acidic by-products which was observed as a fast decrease of pH. No growth was observed after the pH dropped below 5.2. (at 7 h). As demonstrated previously by Tolosa et al. and Wittman et al. [15,17], stops of shaking due to sampling can be observed as rapid decrease of oxygen level (Fig. 2). It is known that already short periods of anaerobic conditions induce unwanted physiological effects like reduced plasmid stability [22,23]. After 9 hours cultivation an increase of the oxygen level was observed. This can be caused either by starvation (due to lack of energy source) or inactivation of respiration activities (due to low pH or cell death). If such culture, even in the exponential growth phase, would be used for inoculation of a bioreactor, bacteria would need much time to adapt to new pH (5 to 7), to change from anaerobic or starvation metabolism to aerobic exponential growth, or to process harmful metabolites in the medium.

Oxygen level monitoring brings new possibilities to adjust cultivation conditions, since anaerobic conditions can be observed and consequently avoided. Although it should be remembered that induction of recombinant gene's expression will severely change the bacteria's metabolic activities, the data in Fig. 2 suggest that standard protocols in small scale recombinant protein production may have a strong physiological background. The cultivation became anaerobic when OD_600 _reached 2.0 (in complex media like LB this happens much earlier, not shown), while OD_600 _values around 0.5 are typically used for induction of recombinant genes. This implies that the oxygen level would be zero already after 2 generations after induction. Additionally, two hours later the pH level dropped below 5.0, which is non-optimal for the growth of *E. coli*.

The stop of cell growth and possible deterioration of recombinant protein quality is not usually caused by depletion of energy source. Instead drop of pH, accumulation of harmful metabolites into the medium and physiological changes due to adaptation of *E. coli *to acidic and anaerobic conditions may be the reason. Monitoring pH changes not only next day but during the cultivation is important, since the pH level may rise again due to assimilation of acetate after glucose consumption. Online monitoring provides a tool to evaluate culture quality and improve culture conditions. By medium design or strain selection (*Escherichia coli *B and K derivatives have different rates of acetate accumulation), medium acidification problems can be reduced.

### Standard substrate feeding protocol for *Pichia pastoris *shake flask cultures is not optimal

**Figure 3:**
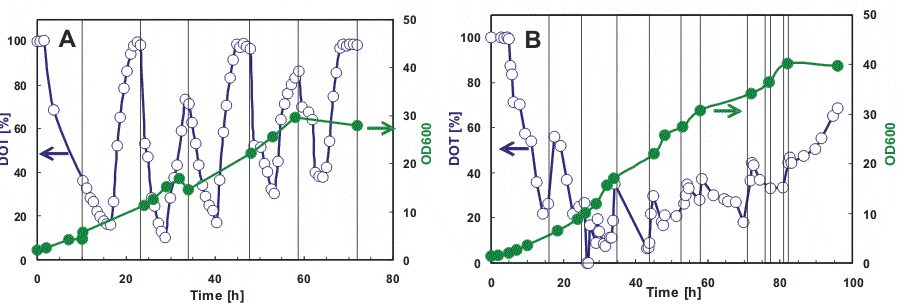
**Optimisation of substrate feeding in *Pichia *pastoris cultures**. *Pichia *uses methanol as a carbon/energy source. Methanol, however, is toxic at high concentrations. A recombinant strain of *Pichia pastoris *GS115 was cultivated in 1 l baffled shake flasks with three side necks containing 200 ml of BMM medium at 30°C with 200 rpm. To maintain the expression of the product, 100 % of methanol was added to a final concentration of 0.5 % (v/v) either twice a day (a) or alternatively, at the time when the DOT level increased (b). Times for substrate feeding are indicated with vertical lines.

*Pichia pastoris *is a metylotrophic yeast which can grow by using methanol as a sole carbon source. This yeast became a widely used host for recombinant protein production. Therefore generally the strong AOX1 promoter is used to control the expression of the target gene, which is induced by methanol. Aside from being the inducer, methanol also serves as the sole carbon and energy source for such cultivations. However, at concentrations above 3 to 5 g l ^-1 ^methanol inhibits the growth [24]. Therefore it is necessary to control the methanol concentration within a small concentration range. The standard methanol feeding strategy according to *Pichia *Expression Kit, Invitrogen, [25] includes methanol feeding to 0.5% (v/v) concentration twice a day. The methanol metabolism requires much oxygen, which can be observed as a fast decrease in oxygen level after methanol addition. High oxygen levels between methanol feedings however indicated that with the generally used standard procedure *Pichia *cells suffer regularly for long time intervals from carbon/energy starvation (Fig. 3a). Consequently we tried to optimize the methanol feeding. Continuous methanol feeding is can not be easily performed in shake flasks due to the very small volumes of methanol which should be added. Therefore we applied a strategy with repeated addition of methanol in dependence on the oxygen signal. When oxygen level in the culture started to increase, indicating exhaustion of methanol, a new methanol pulse up to 0.5 % (v/v) final concentration was added into the medium (Fig. 3b). As a result, 40 % increase in cell density and significantly enhanced mRNA synthesis of product related mRNAs (not shown) were achieved compared to the standard protocol. Currently we are studying the possibilities to improve recombinant collagen production in *Pichia *by optimised feeding. A computer-controlled feeding device which cooperates with the SENBIT system will be applied in further studies to automatically optimise the substrate feed.

## Conclusion

The new wireless system provides pH and dissolved oxygen level monitoring to shake flasks and thereby may be the basis for quality control and optimisation of shake flask cultures. Because such flask cultures can be located in closed chambers and in rotating devices, the wireless measurement with the here presented system offers new flexibility to microbiological or biochemical laboratories. SENBIT however can not, by any means, compete with the possibilities of real bioreactors, since the possibilities to control any of the physical parameters in shake flasks are limited. As demonstrated by *Pichia pastoris *cultivations, some level of process control can be obtained, because the researcher can view the conditions at-time. For example oxygen or substrate limitations can be easily detected and possibly avoided. Reproducibility of shake flask cultivations (cell yield, recombinant protein yield and quality) is usually not satisfactory. Improved measuring possibilities bring the possibility to see what might be the cause of the differences and help to develop better laboratory routines and research protocols. Thereby it is proposed to use the new system in all of the parallel cultures, as the inserted sensors may change the hydrodynamic parameters in the shake flask.

Compared to other small scale cultivation measuring platforms the here presented system has some clear advantages. (i) It is a fully decentralised system, (ii) it does not require separate reader devices, (iii) the distance of data transmission is long enough to allow reading from separate laboratories or cultivation rooms, and (iv) it has a modular design allowing the use of several sensor types and many users. Currently this new system supports pH, oxygen, temperature and pressure sensors. However, because it is not bound to any specific operation principle (like fluorescent sensing), support for other types of sensors is principally possible and currently under development. Beside the use in microbial cultivations, the new wireless platform is also a versatile tool for other measurements in industrial and environmental applications. It has a better mobility than wired systems and new measurements can be set up fast, mostly without changes at the target site. The possibility for such at-time measurements outperforms the use of data-loggers.

## Methods

### Sensors and shake flasks

pH was measured with an autoclavable electrochemical pH sensor (EGV150, Sensortechnik Meinsberg, Germany). Dissolved oxygen was monitoried with a 6 mm diameter polarographic Clark-electrode manufactured by Medorex (Nörten-Hardenberg, Germany). Both sensors were sterilized by autoclavation at 121°C for 20 min before use. 1000 ml 3-baffled shake flasks with three side necks for placement of the electrodes were obtained from Glasgerätebau Ochs (Bovenden, Germany).

### *Escherichia coli *cultivation in mineral salt medium (MSM)

*Escherichia coli *K-12 strain RV308 was cultivated at 37°C in 1 l shake flasks with four vertical baffles containing 200 ml mineral salt medium [26] with 5 gl^-1 ^of glucose as carbon source, 100 mg l^-1 ^thiamine and a starting pH of about 7.0. Overnight cultures were prepared in 100 ml shake flasks with 10 ml of the same medium. For shake flask cultivation the preculture was diluted (OD_600 _= 0.1) into fresh medium.

### *Pichia pastoris *cultivation with methanol feeding

A recombinant strain of *Pichia pastoris *GS115 containing a plasmid for expression of type II human collagen gene cloned under control of the AOX1 promoter (kindly obtained from FibroGen Europe Ltd.) together with propyl-4-hydroxylase encoding gene was cultivated according to Pichia Expression Kit (Invitrogen) instructions [25]. It was first cultivated in a 1 l shake flask with 100 ml buffered minimal medium (BMG) containing 10 g l^-1 ^glycerol. Cells were grown for 14 h at 30°C on a shaker with 200 rpm. When the OD_600 _reached a value between 2 and 6, the cells were centrifuged at 3500 rpm for 5 min, and washed with methanol medium by further centrifugation. The supernatant was decanted and the cell pellet was re-suspended to obtain an initial OD_600 _around 1.5 in a methanol containing medium (buffered minimal methanol medium, BMM). Baffled shake flasks with three side necks containing 200 ml of BMM medium and a pH and an oxygen sensors was incubated at 30°C on a shaker (Certomat^® ^MO, B. Braun Biotech International) with 200 rpm. To maintain the expression of the product, 100% of methanol was added to a final concentration of 0.5% (v/v) twice a day, every 10 to 14 hours, or alternatively, at the time when the DOT level increased

## Authors' contributions

JP carried out *E. coli *cultivations and assisted in all experiments which were monitored with SENBIT^® ^system by setting up and supervising measurements and data analysis. Monika Bollók designed and performed *P. pastoris *cultivation experiments. AV was responsible for software design and programming. The initiative for development of the wireless measuring system came from professor PN, who also actively took part to the planning of experiments. Design and manufacturing of wireless communication devices were made by LI and CH.

## References

[B1] Sanden AM, Prytz I, Tubulekas I, Forberg C, Le H, Hektor A, Neubauer P, Pragai Z, Harwood C, Ward A, Picon A, De Mattos JT, Postma P, Farewell A, Nystrom T, Reeh S, Pedersen S, Larsson G (2003). Limiting factors in Escherichia coli fed-batch production of recombinant proteins. Biotechnol Bioeng.

[B2] Akgun A, Maier B, Preis D, Roth B, Klingelhofer R, Buchs J (2004). A novel parallel shaken bioreactor system for continuous operation. Biotechnology Progress.

[B3] Maier U, Buchs J (2001). Characterisation of the gas-liquid mass transfer in shaking bioreactors. Biochemical Engineering Journal.

[B4] Mrotzek C, Anderlei T, Henzler HJ, Buchs J (2001). Mass transfer resistance of sterile plugs in shaking bioreactors. Biochemical Engineering Journal.

[B5] Yamanè T, Shimizu S, Fiechter A (1984). Fed-batch techniques in microbial processes. Bioprocess parameter control.

[B6] Enfors SO, Häggström L (2005). Bioprocess Technology - Fundamentals and Applications A textbook for introduction of the theory and practice of biotechnical processes.

[B7] Ponce E (1999). Effect of growth rate reduction and genetic modifications on acetate accumulation and biomass yields in Escherichia coli. Journal of Bioscience and Bioengineering.

[B8] Kumar S, Wittmann C, Heinzle E (2004). Minibioreactors. Biotechnol Lett.

[B9] Kostov Y, Harms P, Randers-Eichhorn L, Rao G (2001). Low-cost microbioreactor for high-throughput bioprocessing. Biotechnology and Bioengineering.

[B10] Maharbiz MM, Holtz WJ, Howe RT, Keasling JD (2004). Microbioreactor arrays with parametric control for high-throughput experimentation. Biotechnol Bioeng.

[B11] Szita N, Boccazzi P, Zhang ZY, Boyle P, Sinskey AJ, Jensen KF (2005). Development of a multiplexed microbioreactor system for high-throughput bioprocessing. Lab on A Chip.

[B12] John GT, Klimant I, Wittmann C, Heinzle E (2003). Integrated optical sensing of dissolved oxygen in microtiter plates: a novel tool for microbial cultivation. Biotechnol Bioeng.

[B13] Kensy F, John GT, Hofmann B, Buchs J (2005). Characterisation of operation conditions and online monitoring of physiological culture parameters in shaken 24-well microtiter plates. Bioprocess and Biosystems Engineering.

[B14] Samorski M, Muller-Newen G, Buchs J (2005). Quasi-continuous combined scattered light and fluorescence measurements: A novel measurement technique for shaken microtiter plates. Biotechnology and Bioengineering.

[B15] Tolosa L, Kostov Y, Harms P, Rao G (2002). Noninvasive measurement of dissolved oxygen in shake flasks. Biotechnology and Bioengineering.

[B16] Gupta A, Rao G (2003). A study of oxygen transfer in shake flasks using a non-invasive oxygen sensor. Biotechnol Bioeng.

[B17] Wittmann C, Kim HM, John G, Heinzle E (2003). Characterization and application of an optical sensor for quantification of dissolved O2 in shake-flasks. Biotechnol Lett.

[B18] Guarino RD, Dike LE, Haq TA, Rowley JA, Pitner JB, Timmins MR (2004). Method for determining oxygen consumption rates of static cultures from microplate measurements of pericellular dissolved oxygen concentration. Biotechnol Bioeng.

[B19] Puskeiler R, Kaufmann K, Weuster-Botz D (2005). Development, parallelization, and automation of a gas-inducing milliliter-scale bioreactor for high-throughput bioprocess design (HTBD). Biotechnol Bioeng.

[B20] Stitt DT, Nagar MS, Haq TA, Timmins MR (2002). Determination of growth rate of microorganisms in broth from oxygen-sensitive fluorescence plate reader measurements. Biotechniques.

[B21] Wittmann C, Kim HM, John G, Heinzle E (2003). Characterization and application of an optical sensor for quantification of dissolved O-2 in shake-flasks. Biotechnology Letters.

[B22] Hopkins DJ, Betenbaugh MJ, Dhurjati P (1987). Effects of Dissolved-Oxygen Shock on the Stability of Recombinant Escherichia-Coli Containing Plasmid Pkn401. Biotechnology and Bioengineering.

[B23] De Leon A, Hernandez V, Galindo E, Ramirez OT (2003). Effects of dissolved oxygen tension on the production of recombinant penicillin acylase in Escherichia coli. Enzyme and Microbial Technology.

[B24] Mayson BE, Kilburn DG, Zamost BL, Raymond CK, Lesnicki GJ (2003). Effects of methanol concentration on expression levels of recombinant protein in fed-batch cultures of Pichia methanolica. Biotechnology and Bioengineering.

[B25] Invitrogen (2005). Pichia Expression Kit, Protein Expression, A Manual of Methods for Expression of Recombinant Proteins in Pichia pastoris. Catalog no. K1710-01.. http://www.invitrogen.com.

[B26] Neubauer P, Haggstrom L, Enfors SO (1995). Influence of Substrate Oscillations on Acetate Formation and Growth-Yield in Escherichia-Coli Glucose-Limited Fed-Batch Cultivations. Biotechnology and Bioengineering.

